# Exoskeleton Hand Control by Fractional Order Models

**DOI:** 10.3390/s19214608

**Published:** 2019-10-23

**Authors:** Mircea Ivanescu, Nirvana Popescu, Decebal Popescu, Asma Channa, Marian Poboroniuc

**Affiliations:** 1 Department of Mechatronics, University of Craiova, 200585 Craiova, Romania; 2 Department of Computer Science, University Politehnica of Bucharest, 060042 Bucharest, Romania; nirvana.popescu@cs.pub.ro (N.P.); decebal.popescu@cs.pub.ro (D.P.); asma.channa@admin.muet.edu.pk (A.C.); 3 Department of Electrical Engineering, Technical University of Iasi, 700050 Iași, Romania; mpobor@tuiasi.ro

**Keywords:** exoskeleton hand, fractional order model, control

## Abstract

This paper deals with the fractional order control for the complex systems, hand exoskeleton and sensors, that monitor and control the human behavior. The control laws based on physical significance variables, for fractional order models, with delays or without delays, are proposed and discussed. Lyapunov techniques and the methods that derive from Yakubovici-Kalman-Popov lemma are used and the frequency criterions that ensure asymptotic stability of the closed loop system are inferred. An observer control is proposed for the complex models, exoskeleton and sensors. The asymptotic stability of the system, exoskeleton hand-observer, is studied for sector control laws. Numerical simulations for an intelligent haptic robot-glove are presented. Several examples regarding these models, with delays or without delays, by using sector control laws or an observer control, are analyzed. The experimental platform is presented.

## 1. Introduction

The IHRG is an intelligent haptic robotic glove system for the rehabilitation of patients that have a diagnosis of a cerebrovascular accident. This system is created by a thin textile in order to have a comfortable environment for grasping exercises. An exoskeleton architecture ensures the mechanical compliance of human fingers. The driving and skin sensor system is designed to determine comfortable and stable grasping function. This paper analyzes the dynamics of an exoskeleton hand using fractional order operators and proposes control solutions.

The number of applications in the system modelling, where the fractional order calculus (FOC) is used, has increased significantly in the last few decades. Many authors proved that non-integer order integrals and derivatives are suitable for analyses of the properties of various materials. Recent achievements in the interpretation of FOC operators allowed to apply FOC for processes that are better described by fractional order models (FOM) rather than integer order models (IOM). The role of these models in soft matter physics and viscoelastic behavior, in the theory of complex materials, its quality to include effects with non-conservative forces and power-law phenomena suggest to describe the complexity of human dynamics using FOM operators [1].

The idea is supported by the evidence of $s^{\beta }$ dynamics in muscles and joint tissues throughout human musculo-skeletal system [2,3]. Interaction and dependence between biological systems and associated mechanical components was analyzed in [4,5,6]. Fractional order models for metal polymer composite was discussed in [7]. In [8,9] viscoelastic properties for a large variety of biological entities were studied. A class of sensors based on fractional calculus was presented in [10]. Optimal techniques using fractional calculus for sensor networks were discussed in [11]. A fractional model to capture muscular dynamics in the movement process was proposed in [12]. In [13,14,15] a class of neural, muscular, and vascular processes were studied to minimize sensor placement. Recently, there is a great deal of interest in so-called rehabilitation robotics, a branch of the areas of robotics and mechatronics that addresses the study of complex robotic systems aiming to restore human functions for those who suffer major trauma as a result of strokes and cerebrovascular accidents. Robotic therapy is a promising form of neurorehabilitation that can be delivered in more intensive regimens compared to conventional therapy [16]. The complexity of these systems associated with a specified class of sensors is well described by fractional order differential equations [17,18]. The methods for the analysis and design of fractional order operators can be found in [19]. Control and stability of FOM was investigated by techniques Lyapunov in [20,21]. In particular, the authors of [22,23] discuss the stability properties of solutions of nonlinear Caputo fractional differential equations. The exponential stability of nonlinear FOM using the Lyapunov method was analyzed in [24,25]. Other control problems for a class of FOMs with delay were rigorously investigated in [26,27]. Reference [28] proposed an observer for a class of linear and nonlinear FOM using Lyapunov methods. To our knowledge, this paper is the first paper to assess FOM for systems and sensors that monitor or control human behavior. The exoskeleton architecture, which ensures a mechanical compliance of human fingers, including the driving and sensor system, determines comfortable and stable grasping functions.

The dynamics of the whole system, exoskeleton hand (EXHAND), and the sensors can be accurately described by FOM operators. A class of 3D FOM bending sensors is analyzed. The control laws based on physical significance variables, for linear and delay FOM or IOM systems, are proposed and discussed. The sector control laws for linear FOM, with delays or without delays are studied. Lyapunov techniques and the methods that derive from Yakubovici-Kalman-Popov Lemma are used, and the frequency criteria that ensure asymptotic stability of the physical significance variable closed loop system are inferred. An observer control is proposed for the complex models, EXHANDs and sensors. The asymptotic stability of the whole system, the observer-system, is studied according to sector control laws. Frequency criteria and conditions for asymptotic stability are determined. Numerical simulations for the intelligent haptic robot-glove (IHRG) are presented. Several examples regarding the FOM or IOM systems, with delays or without delays, by using sector control laws or an observer control, are analyzed. The IHRG experimental platform is then discussed.

The paper is structured as follows: Section 2.1 discusses FOM sensors and FOM systems implemented in EXHAND; Section 2.2 presents the control systems; Section 3.1 analyzes IHRG numerical simulations; and Section 3.2 presents the IHRG platform. Section 4 provides concluding remarks and discussions.

## 2. Methods

### 2.1. Fractional Order Models

Notations:

1. The fractional order integral of order β is the Riemann-Liouville fractional integral:
  $$I^{\beta }=\frac{1}{\Gamma \left(\beta \right)}\overset{t}{\underset{0}{\int }}f\left(\theta \right){\left(t-\theta \right)}^{\beta -1}d\theta$$
2. The Caputo derivative of order β, 0< β<1 is:
  $$D^{\beta }f\left(t\right)=\frac{1}{\Gamma \left(\beta -1\right)}\overset{t}{\underset{0}{\int }}\dot{f}\left(\theta \right){\left(t-\theta \right)}^{-\beta }d\theta$$
  where β is the fractional order exponent and Γ(β) is the gamma (Euler’s) function.

(a) 3D curvature sensors described by FOM

**Figure 1:**
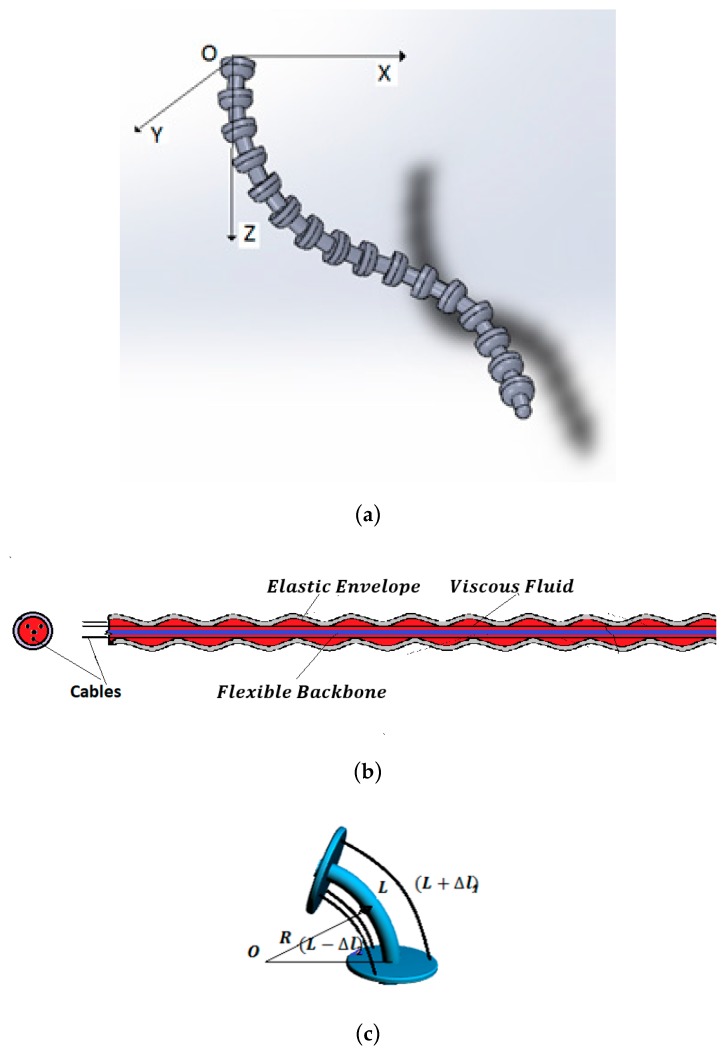
(**a**) 3D hyper-redundant robotic arm. (**b**) 3D FOM curvature sensor. (**c**) Measurement technique.

Bending sensors represent a class of sensors with large applications in the control of complex systems. They convert changes in bend to an electrical parameter variation. Conventional bending sensors handle cases in which bending is produced in the 2D plane. The most common are the resistive sensors, described by IOM operators of order 0. For a special class of systems, such as the hyper-redundant robots [29] where bending is produced in a 3D space (Figure 1a), a special class of bending sensors defined by FOM operators (Figure 1b) is used.

The architecture of this sensor consists of a main viscoelastic component determined by a long flexible backbone wrapped in a cylindrical elastic envelope. Three antagonist cables are implemented at the periphery of the system. In static behavior, curvature κ is obtained by the differential measurement of the cable lengths, Figure 1c [30]:

(1) $$\kappa =F\left(\Delta L_{1},\Delta L_{2},\;\Delta L_{3}\right)$$

**Figure 2:**
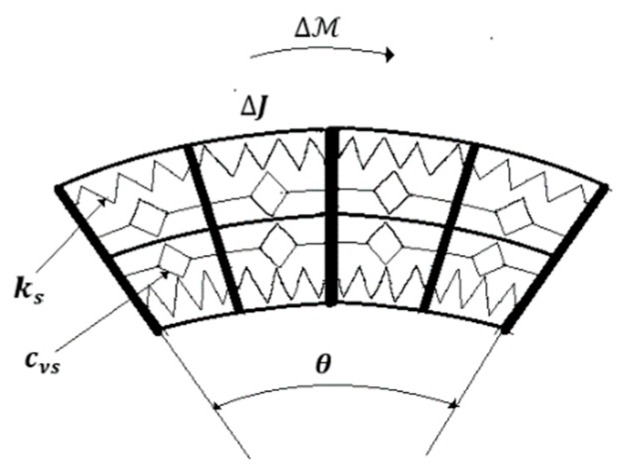
Technological equivalent model of the 3D curvature sensor.

The dynamic behavior is inferred considering constant curvature along the length. Employing the same technique as that developed in [17] yields (Figure 2):

(2) $$\ddot{\kappa }\left(t\right)=-c_{\nu s}b_{s}D^{\beta }\kappa \left(t\right)-k_{s}c_{s}\kappa \left(t\right)+k_{M}ℳ\left(t\right)$$

where $c_{\nu s},\;k_{s}$ are distributed viscous and elastic coefficient, assumed uniform distributed along the length, $b_{s},\;c_{s}$ are material parameters and ℳ is the moment that determines the bending. The transfer function is derived from Equation (2) as:

(3) $$H_{s}\left(s\right)=\frac{\kappa \left(s\right)}{ℳ\left(s\right)}=\frac{k_{M}}{s^{2}+c_{\nu s}b_{s}s^{\beta }+k_{s}c_{s}}$$

That corresponds to an order 2 FOM operator.

(b) FOM systems

**Figure 3:**
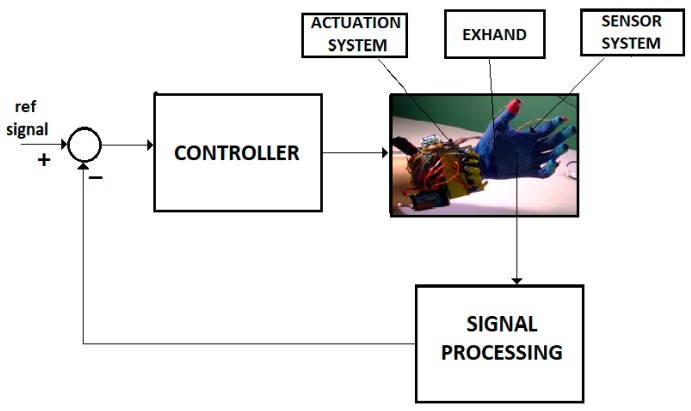
IHRG system.

A large class of systems that monitors or controls the human behavior is well described by the FOM operators. Figure 3 shows the control system of an intelligent haptic robot-glove (IHRG) for the rehabilitation of patients that have a diagnosis of a cerebrovascular accident. The IHRG is a medical device that acts in parallel to a hand in order to compensate for lost functions [16]. The exoskeleton architecture that ensures the mechanical compliance of human fingers for the driving system determines comfortable and stable grasping functions.

The dynamics of the system (EXHAND) can be accurately described by FOM operators,

(4) $$D^{\beta }z\left(t\right)=A_{0}z\left(t\right)+f\left(z\right)+bu\left(t\right),\;t\in \left[0,\;T\right]$$

where z is the state vector $z={\left[z_{1},\;z_{2},\ldots ,z_{n}\right]}^{T}$ that defines the motion parameters, β is the fractional order exponent, and $A_{0},\;b$ are (n×n), (n×1) constant matrices. In a FOM operator of EXHAND, the vector components are defined as

(5) $$D^{\beta }z_{1}=z_{2},\;D^{\beta }z_{2}=z_{3},\ldots$$

The nonlinear term f(z) is determined by the gravitational components and satisfies the inequality

(6) ||f(z)||<η||z||

The output of the system is generated by the bending sensors. Provided that the bending of the phalange musculoskeletal system is in 2-D plane, in this project, we used an Arduino Flex Resistive Sensor network. This sensor operates as a zeroth IOM operator,

(7) $$y\left(t\right)=c^{T}z\left(t\right)$$

where c is a constant (n×1) vector.

A new model can be inferred if the delay time constant, associated with the neuro-muscular system, the driving system and the processing time, is introduced,

(8) $$D^{\beta }z\left(t\right)=A_{0}z\left(t\right)+A_{1}z\left(t-\tau \right)+f\left(z\right)+bu\left(t\right),\;t\in \left[0,\;T\right]$$

The initial conditions are defined by

z(t)=φ(t),  t∈[−τ, 0]

where the function φ is associated to initial states.

**Figure 4:**
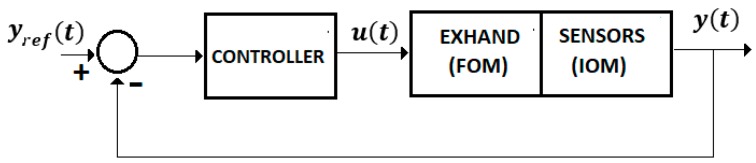
Control system.

For Equations (4)–(8) we used the control system from Figure 4 with a FOM operator for the EXHAND and a IOM operator for the sensor system.

### 2.2. Control Systems

Mathematical Preliminaries

**Lemma**  [19]**.** *For any symmetric matrix* $P\in R^{nxn}$ *, the following inequality holds:*

(9) $$\lambda_{\min\left(P\right)}I^{*}\;\leq P\;\leq \;\lambda_{\max\left(P\right)}I^{*}$$

*where* $\lambda_{min\left(P\right)},\;\lambda_{max\left(P\right)}$ *denote the minimum and maximum eigenvalue, respectively, of matrix* P *and* $I^{*}$ *is the unit matrix.*

**Theorem 1**  ([21,22,24])**.** *The system* $D^{\beta }z\left(t\right)=Az\left(t\right),\;0<\beta <1$*, is asymptotically stable if*

(10) $$\left|Arg\left(eig\left(A\right)\right)\right|>\beta \frac{\pi }{2}$$

**Theorem 2**  ([22,23,24])**.** *The system* $D^{\beta }z\left(t\right)=f\left(z\left(t\right)\right),\;z\left(t_{0}\right)=z_{0}$ *is asymptotically stable if there exists a continuously differentiable function* V(t, z) *that satisfies*

(11) $$\alpha_{1}{\left||z|\right|}^{2}\;\leq V\left(t,\;z\left(t\right)\right)\leq \;\alpha_{2}{\left||z|\right|}^{2}$$

(12) $$D^{\beta }V\left(t,\;z\left(t\right)\right)\leq \;-\alpha_{3}{\left||z|\right|}^{2}$$

*where* $\alpha_{1},\;\alpha_{2},\;\alpha_{3}$ *are positive constants,*

0<β<1.

#### 2.2.1. Control for the EXHAND Without Delays

Consider the system from Figure 4 defined by Equations (4)–(7) without a delay time. Assume a control law.

(13) $$u\left(t\right)=-k\left(y\left(t\right)-y_{ref}\left(t\right)\right)$$

where the control gain *k* verifies the condition

(14) kσ ≤1

where σ is a positive constant (for simplicity, $y_{ref}\left(t\right)=0$).

**Remark** **1.** *For the EXHAND model with the state variables defined by Equation (5) and the output vector* $c={\left[c_{1}.\;c_{2},\;c_{3},\;c_{4}\right]}^{T}$ *, the control law (Equation (10)) becomes a* $PD^{\beta }$ *law*

$$u\left(t\right)=-k\left(c_{1}z_{1}+c_{2}D^{\beta }z_{1}+c_{3}z_{3}+c_{4}D^{\beta }z_{3}\right)$$

*or*

(15) $$u\left(t\right)=-k_{1}z_{1}-D^{\beta }z_{1}-k_{3}z_{3}-k_{4}D^{\beta }z_{3}$$

*If c is selected as*$c={\left[c_{1}.\;0,\;c_{3},\;0\right]}^{T}$, *the control becomes a PD law*

(16) $$u\left(t\right)=-k_{1}z_{1}-k_{3}{\dot{z}}_{1}$$

**Control** **System** **1.** The system (Equations (4)–(7)) with the controller defined by Equations (13) and (14) is asymptotically stable if:

The matrix $A^{*}=A-R$ is Hurwitz stable where $R=R^{T}>0$.

(17) $$Re\left(c^{T}{\left(j\omega I-\left(A-R\right)\right)}^{-1}\;b\right)\;\geq \;-\sqrt{\sigma }$$

(18) $$\varrho >\;\rho_{PR}+2\lambda_{\max\left(P\right)}\eta$$

where $\varrho =||\left(q+k\sqrt{\sigma }d\right){\left(q+k\sqrt{\sigma }d\right)}^{T}||$, $\rho_{PR}=2PR$ and $Q=qq^{T}$, P are solutions of the Lyapunov equation [20,29].

**Proof.**  Consider the Lyapunov function

(19) $$V\left(z\right)=\;z^{T}Pz$$

where $P=P^{T}>0$. The first asymptotic stability conditions (Equation (8)) are verified for $\alpha_{1}=\lambda_{\min\;\left(P\right)},\;\alpha_{2}=\lambda_{\max\;\left(P\right)}$, [21] (Theorem 1), where $\lambda_{\min\;\left(P\right)},\;\lambda_{\max\;\left(P\right)}\;$ denote the minimum and maximum eigenvalues of P. The fractional derivative of Equation (14) will be [22,24],

(20) $$D^{\beta }V\left(z\right)\leq \left(D^{\beta }z^{T}\right)Pz+\;z^{T}P\left(D^{\beta }z\right)$$

By substituting Equation (4) into Equation (20), one derives

(21) $$D^{\beta }V\left(z\right)\leq \;z^{T}\left(A^{T}P+PA\right)z+2z^{T}Pbu+2z^{T}Pf$$

Employing the condition (a) yields

(22) $$D^{\beta }V\left(z\right)\leq \;z^{T}\left({\left(A-R\right)}^{T}P+P\left(A-R\right)\right)z+2z^{T}Pbu+\;2z^{T}PRz+2z^{T}Pf$$

Considering Equations (13) and (14), this inequality becomes

(23) $$D^{\beta }V\left(z\right)\leq \;z^{T}\left({\left(A-R\right)}^{T}P+P\left(A-R\right)\right)z+2z^{T}\left(Pb-\frac{1}{2}c\right)u-\sigma u^{2}+\;2z^{T}PRz+2z^{T}Pf$$

By employing the condition (Equation (15)) and Yakubovici-Kalman-Popov (YKP) Lemma [31], results

(24) $$z^{T}\left({\left(A-R\right)}^{T}P+P\left(A-R\right)\right)z=-qq^{T}$$

(25) $$Pb-\frac{1}{2}c=\sqrt{\sigma }q$$

Now, considering the control law (Equation (13)), it follows that

(26) $$D^{\beta }V\left(z\right)\leq -z^{T}\left(q+k\sqrt{\sigma }d\right){\left(q+k\sqrt{\sigma }d\right)}^{T}z+\rho_{PR}z^{T}z+2\lambda_{\max\left(P\right)}\eta$$

or, by Equation (16),

(27) $$D^{\beta }V\left(z\right)\leq -\alpha_{3}{\left||z|\right|}^{2}$$

where

(28) $$\alpha_{3}=\varrho -\rho_{PR.\;\;}$$

 □

#### 2.2.2. Control for the EXHAND with Delay

**Control** **System** **2.** The system described by Equation (8) with the control law defined by Equation (13) is asymptotically stable if:

1.  $A_{0}^{*}=\left(A_{0}-R\right)$ is Hurwitz stable, where $R=R^{T}>0$

(29) $$Re\left(c^{T}{\left(j\omega I-A_{0}^{*}\right)}^{-1}b\right)\geq -\sqrt{\sigma }$$

(30) $$\varrho -\left(2\eta \lambda_{\max\left(P_{1}\right)}+\frac{1}{2}\lambda_{\max\left(D\right)}+2\lambda_{\max\left(P_{1}R\right)}+\lambda_{\max\left(P_{2}\right)}\right)>0$$

(31) $$\lambda_{\min\left(P_{2}\right)}-\frac{1}{2}\lambda_{\max\left(D\right)}>0$$

where $Q=qq^{T}$, $P_{1}$ are solutions of the Lyapunov equations and

$$\varrho =||\left(q+k\sqrt{\sigma }c_{1}\right){\left(q+k\sqrt{\sigma }c_{1}\right)}^{T}||$$

$$D=\left(A_{1}^{T}P_{1}+P_{1}A_{1}\right)$$

**Proof.**  Consider the following Lyapunov function:

(32) $$V\left(z\left(t\right)\right)=I^{1-\beta }\left(z^{T}\left(t\right)P_{1}z\left(t\right)\right)+\overset{t}{\underset{t-\tau }{\int }}z^{T}\left(\theta \right)P_{2}z\left(\theta \right)d\theta$$

where $P_{1},\;P_{2}$ are (n×n) are positive definite and symmetrical matrices, $P_{1}>0,\;P_{2}>0,\;P_{1}^{T}=P_{1},P_{2}^{T}=P_{2}$. V(z) satisfies the condition (Equation (11)) of Theorem 2.

(33) $$V\left(z\right)\geq \lambda_{\min\left(P_{1}\right)}{\left||z\left(t\right)|\right|}^{2}$$

(34) $$V\left(z\right)\leq \lambda_{\max\left(P_{1}\right)}{\left||z\left(t\right)|\right|}^{2}+\lambda_{\max\left(P_{2}\right)}\overset{t}{\underset{t-\tau }{\int }}{\left||z\left(\theta \right)|\right|}^{2}d\theta \leq M{\left||z\left(t\right)|\right|}^{2}$$

The derivative $D^{\beta }V\left(z\right)$ is computed from:

(35) $$D^{\beta }V\left(z\right)=I^{1-\beta }\dot{V}\left(z\right)$$

where $I^{1-\beta }$ is the Riemann-Liouville fractional integral of the order (1−β). The derivative $\dot{V}\left(z\right)$ is evaluated from Equation (32)

(36) $$\dot{V}\left(z\right)=D^{\beta }(\left(z^{T}\left(t\right)P_{1}z\left(t\right)\right)+\frac{d}{dt}\overset{t}{\underset{t-\tau }{\int }}z^{T}\left(\theta \right)P_{2}z\left(\theta \right)d\theta$$

which leads to the inequality

(37) $$\dot{V}\left(z\right)\leq (D^{\beta }z^{T}\left(t\right))P_{1}z\left(t\right)+z^{T}\left(t\right)P_{1}\left(D^{\beta }z\left(t\right)\right)+z^{T}\left(t\right)P_{2}z\left(t\right)-z^{T}\left(t-\tau \right)P_{2}z\left(t-\tau \right)$$

By evaluating Equation (37) along of solutions of Equation (8) it turns out that

(38) $$\dot{V}\left(z\right)\leq z^{T}\left(t\right)\left(A_{0}^{*T}P_{1}+P_{1}A_{0}^{*}\right)z\left(t\right)+z^{T}\left(t\right)\left(A_{1}^{T}P_{1}+P_{1}A_{1}\right)z\left(t-\tau \right)+\;2z^{T}\left(t\right)P_{1}bu\left(t\right)+2z^{T}Pf+2z^{T}\left(t\right)P_{1}Rz\left(t\right))+z^{T}\left(t\right)P_{2}z\left(t\right)-z^{T}\left(t-\tau \right)P_{2}z\left(t-\tau \right)$$

By applying the control law Equation (28), it yields

(39) $$\dot{V}\left(z\right)\leq z^{T}\left(t\right)\left(A_{0}^{*T}P_{1}+P_{1}A_{0}^{*}\right)z\left(t\right)+2z^{T}\left(t\right)(P_{1}b-\frac{c}{2})u\left(t\right)-\sigma u^{2}\left(t\right)+2z^{T}Pf+z^{T}\left(t\right)\left(A_{1}^{T}P_{1}+P_{1}A_{1}\right)z\left(t-\tau \right)+2z^{T}\left(t\right)P_{1}Rz\left(t\right)+z^{T}\left(t\right)P_{2}z\left(t\right)-z^{T}\left(t-\tau \right)P_{2}z\left(t-\tau \right)$$

The following inequality will be used [23]

(40) $$||z^{T}\left(t\right)Dz\left(t-\tau \right)||\leq ||z\left(t\right)||\;||D||\;||z\left(t-\tau \right)||\leq \lambda_{\max\left(D\right)}\left(\frac{{\left||z\left(t\right)|\right|}^{2}}{2}+\frac{{\left||z\left(t-\tau \right)|\right|}^{2}}{2}\right)$$

Additionally, considering the YKP Lemma as in the previous Control System, yields

(41) $$z^{T}\left(\left(A^{*}-R{)}^{T}P_{1}+P_{1}(A^{*}-R\right)\right)z=-qq^{T}$$

(42) $$P_{1}b-\frac{1}{2}c=\sqrt{\sigma }q$$

Substituting this result into Equation (39), considering the inequalities of Equations (6) and (40), one derives that

(43) $$\begin{array}{cc} \dot{V}\left(z\right)\leq & -z^{T}\left(t\right)\left(q+k\sqrt{\sigma }d\right){\left(q+k\sqrt{\sigma }d\right)}^{T}z\left(t\right)\\ & +\left(2\eta \lambda_{\max\left(P_{1}\right)}+\frac{1}{2}\lambda_{\max\left(D\right)}+2\lambda_{\max\left(P_{1}R\right)}+\;\lambda_{\max\left(P_{2}\right)}\right){\left||z\left(t\right)|\right|}^{2}\\ & -\left(\lambda_{\min\left(P_{2}\right)}-\frac{1}{2}\lambda_{\max\left(D\right)}\right){\left||z\left(t-\tau \right)|\right|}^{2} \end{array}$$

Employing Equations (30) and (31), yields

(44) $$\dot{V}\left(z\right)\leq -\left(\varrho -\left(2\eta \lambda_{\max\left(P_{1}\right)}+\frac{1}{2}\lambda_{\max\left(D\right)}+2\lambda_{\max\left(P_{1}R\right)}+\lambda_{\max\left(P_{2}\right)}\right)\right){\left||z\left(t\right)|\right|}^{2}$$

Denoted by

$$\alpha_{3}=\varrho -(2\eta \lambda_{\max\left(P_{1}\right)}+\frac{1}{2}\lambda_{\max\left(D\right)}+2\lambda_{\max\left(P_{1}R\right)}+\lambda_{\max\left(P_{2}\right)}$$

and from Equation (35) results

(45) $$D^{\beta }V\left(z\right)\leq -\alpha_{3}z{\left(t\right)}^{2}.$$

 □

#### 2.2.3. Control System with Observer for the EXHAND with Delay

Consider the linearized model of Equation (8) rewritten as

(46) $$D^{\beta }z\left(t\right)=A_{L}z\left(t\right)+A_{1}z\left(t-\tau \right)+bu\left(t\right),\;t\in \left[0,\;T\right]$$

(47) $$y\left(t\right)=c^{T}z\left(t\right)$$

where the nonlinear term was approximated by

(48) $$f\left(z\right)≅{\frac{\partial f\left(z\right)}{\partial z}|}_{z_{0}}\Delta z={\frac{\partial f\left(z\right)}{\partial z}|}_{z_{0}}\left(z_{1}-z_{0}\right)={\frac{\partial f\left(z\right)}{\partial z}|}_{z_{0=0}}z_{1}$$

**Remark** **2.** *For the EXHAND model, the pair (* $A_{L},\;b)$ *is controllable and the pair* $\;\left(C,\;A_{L}\right)$ *is observable.*

Consider the system defined by Equation (46). The following observer is proposed:

(49) $$D^{\beta }\hat{z}\left(t\right)=A_{L}\hat{z}\left(t\right)+A_{1}\hat{z}\left(t-\tau \right)+bu\left(t\right)+L_{1}\left(y_{1}\left(t\right)-{\hat{y}}_{1}\left(t\right)\right)+L_{2}\left(y_{2}\left(t-\tau \right)-{\hat{y}}_{2}\left(t-\tau \right)\right)$$

(50) $$\hat{z}\left(t\right)=\hat{\varphi }\left(t\right),t\in \left[-\tau ,0\right]$$

(51) $$\hat{y}\left(t\right)={\left[{\hat{y}}_{1}\left(t\right)\;{\hat{y}}_{2}\left(t\right)\right]}^{T},\;\;\;{\hat{y}}_{1}\left(t\right)=c_{1}^{T}\hat{z}\left(t\right),\;\;{\hat{y}}_{2}\left(t\right)=c_{2}^{T}\hat{z}\left(t-\tau \right)$$

where $\hat{z}\in R^{n}$ is the observer state, $\hat{y}\in R^{2}$ is the estimated output and $L_{1},L_{2}$ are (n×1) observability vectors. The observer error is

(52) $$\Delta z\left(t\right)=z\left(t\right)-\hat{z}\left(t\right)$$

defined by the following equation:

(53) $$D^{\beta }\left(\Delta z\left(t\right)\right)=\left(A_{L}-L_{1}c_{1}^{T}\right)\Delta z\left(t\right)+\left(A_{1}-L_{2}c_{2}^{T}\right)\Delta z\left(t-\tau \right)$$

(54) Δz(t)=Δφ,   t∈[−τ,0]

Consider the control law

(55) $$u\left(t\right)=u_{1}\left(t\right)+u_{2}\left(t\right)=-k_{1}c_{1}^{T}\hat{z}\left(t\right)-k_{2}c_{2}^{T}\hat{z}\left(t-\tau \right)$$

The global state $\left(\hat{z},z-\hat{z}\right)=\;\left(\hat{z},\Delta z\right)\;$ is considered for the system “EXHAND-observer”.

**Figure 5:**
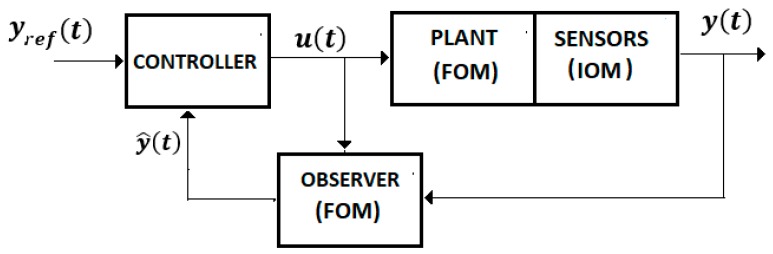
Control system with observer.

**Control** **System** **3.** The whole system, “EXHAND-observer”, Equations (46), (47), and (49)–(51) (Figure 5) with the control law (55), is asymptotically stable if

$A_{L}^{*}=\left(A_{L}-R\right)$ is Hurwitz stable where $R=R^{T}>0.$

(56) $$k_{1}\sigma \leq 1,\;\;\;\sigma >0$$

(57) $$Re\left(c_{1}^{T}{\left(j\omega I-A_{L}^{*}\right)}^{-1}b\right)\geq -\sqrt{\sigma }q$$

(58) $$-\varrho +\lambda_{\max\left(D_{1}\right)}+\lambda_{\max\left(D_{2}\right)}+2\lambda_{\max\left(P_{1}R\right)}+\lambda_{\max\left(P_{3}\right)}<0$$

(59) $$\lambda_{\max\left(E_{1}\right)}-\frac{1}{2}\lambda_{\max\left(D_{1}\right)}-\lambda_{\max\left(P_{2}\right)}>0$$

(60) $$\lambda_{\max\left(P_{2}\right)}-\frac{1}{2}\lambda_{\max\left(E_{2}\right)}>0$$

(61) $$\lambda_{\max\left(P_{3}\right)}-\lambda_{\max\left(D_{2}\right)}>0$$

where ϱ is defined by Equation (50) and

(62) $$D_{2}={\left(Lc_{1}^{T}\right)}^{T}P_{1}+P_{1}Lc_{1}^{T};\;\;\;D_{1}=\left(A_{1}^{T}P_{1}+P_{1}A_{1}\right)$$

(63) $$E_{1}=A_{0}^{*}-L_{1}c_{1}^{T};\;\;\;E_{2}=A_{1}-L_{2}c_{2}^{T}$$

**Proof.**  Consider the Lyapunov function

(64) $$V\left(\hat{z},\;\Delta z\right)=I^{1-\beta }\left({\hat{z}}^{T}\left(t\right)P_{1}\hat{z}\left(t\right)+\frac{1}{2}\Delta z^{T}\left(t\right)\Delta z\left(t\right)\right)+\overset{t}{\underset{t-\tau }{\int }}\left(\Delta z^{T}\left(\theta \right)P_{2}\Delta z\left(\theta \right)+z^{T}\left(\theta \right)P_{3}z\left(\theta \right)\right)d\theta$$

where $P_{1},\;P_{2},\;P_{3}$ are (n×n) are positive definite and symmetrical matrices. V(z, Δz) satisfies the first condition (Equation (11)) of Theorem 2.

Applying the same procedures as in the previous control system, yields

(65) $$\begin{array}{cc} \dot{V}\left(\hat{z},\;\Delta z\right)\leq & -{\hat{z}}^{T}\left(t\right)(\left(q+k_{1}\sqrt{\sigma }c_{1}\right){\left(q+k_{1}\sqrt{\sigma }c_{1}\right)}^{T}\hat{z}\left(t\right)\\ & +\left(\lambda_{\max\left(D_{1}\right)}+\lambda_{\max\left(D_{2}\right)}+2\lambda_{\max\left(P_{1}R\right)}+\lambda_{\max\left(P_{3}\right)}\right){\left||\hat{z}\left(t\right)|\right|}^{2}-(\lambda_{\max\left(E_{1}\right)}-\frac{1}{2}\lambda_{\max\left(D_{1}\right)}\\ & -\lambda_{\max\left(P_{2}\right)})\;{\left||\Delta z\left(t\right)|\right|}^{2}-\left(\lambda_{\max\left(P_{2}\right)}-\frac{1}{2}\lambda_{\max\left(E_{2}\right)}\right){\left||\Delta z\left(t-\tau \right)|\right|}^{2}\\ & -\left(\lambda_{\max\left(P_{3}\right)}-\lambda_{\max\left(D_{2}\right)}\right){\left||\hat{z}\left(t-\tau \right)|\right|}^{2} \end{array}$$

By employing conditions (58)–(61) this inequality becomes

(66) $$\dot{V}\left(\hat{z},\;\Delta z\right)\leq -\left(\varrho -\lambda_{\max\left(D_{1}\right)}-\lambda_{\max\left(D_{2}\right)}-2\lambda_{\max\left(P_{1}R\right)}-\lambda_{\max\left(P_{3}\right)}\right){\left||\hat{z}\left(t\right)|\right|}^{2}-(\lambda_{\max\left(E_{1}\right)}-\frac{1}{2}\lambda_{\max\left(D_{1}\right)}-\lambda_{\max\left(P_{2}\right)})\;{\left||\Delta z\left(t\right)|\right|}^{2}$$

and using (53) yields

(67) $$D^{\beta }V\left(\hat{z},\;\Delta z\right)\leq -\alpha_{3}{\left||\begin{array}{c} \hat{z}\left(t\right)\\ \Delta z\left(t\right) \end{array}|\right|}^{2}.$$

 □

**Remark** **3.** *The asymptotical stability conditions of Control System 2, Control System 3 are independent by the time delay* τ.

## 3. Results

### 3.1. IHRG Control—Numerical Simulations

#### 3.1.1. EXHAND with Sensors Without Delays

Consider the IHRG system of Figure 3. The exoskeleton drive system is a decoupled one, for each finger. The following parameters of the hand and exoskeleton mechanical architecture [16] will be used: the equivalent moment of inertia is $J=0.005\;\mathrm{kg}\cdot m^{2}$, the equivalent mass is m=0.015 kg, the viscous and elastic coefficients of the equivalent Kelvin-Voigt model of the joint tissues throughout phalange musculoskeletal system and exoskeleton are [6,7] $c_{\nu }=0.22\;\mathrm{Nm}\cdot s\cdot {\mathrm{rad}}^{-1}\;c_{e}=2.8\;\mathrm{Nm}\cdot {\mathrm{rad}}^{-1}$, respectively, and the damping coefficient is $c_{d}=7.8\;\mathrm{Nm}\cdot s\cdot {\;\mathrm{rad}}^{-1}$.

(68) $$J\;\ddot{\theta }\left(t\right)=-c_{\nu }\;D^{\beta }\theta \left(t\right)-c_{e}\;\theta \left(t\right)-c_{d}\;\dot{\theta }\left(t\right)+mg\sin\theta +bu\left(t\right)$$

(69) $$\theta \left(0\right)=\left[\frac{\pi }{3},\;0\right]$$

where the nonlinear component verifies the inequality (Equation (6)) for η=0.2. The sensor is considered as an IOM operator and the output is defined as

(70) $$y\left(t\right)=c^{T}\theta \left(t\right)$$

The fractional order exponent is $\beta =\frac{1}{2}$. The FOM model (Equations (8) and (9)) is defined as $\theta_{1}=\theta ;\;D^{\frac{1}{2}}\theta_{1}=\theta_{2};\;D^{\frac{1}{2}}\theta_{2}=\theta_{3}=\dot{\theta };\;D^{\frac{1}{2}}\theta_{3}=\theta_{4}$; $A=\left[\begin{array}{c} 0\;1\;0\;0\\ 0\;0\;1\;0\\ 0\;0\;0\;1\\ -0.6\;-0.1\;-7.8\;0 \end{array}\right]$; $b=\left[\begin{array}{c} 0\\ 0\\ 0\\ 4.5 \end{array}\right]$; $c=\left[\begin{array}{c} 1\\ 0\\ 1\\ 0 \end{array}\right]$.

**Figure 6:**
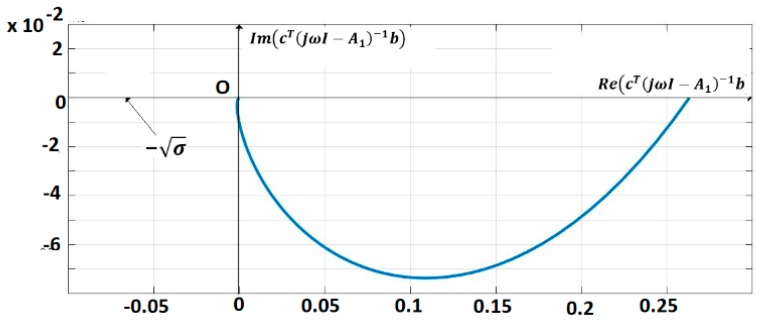
Polar plot of $c^{T}{\left((j\omega I-A_{1}\right)}^{-1}b.$

**Figure 7:**
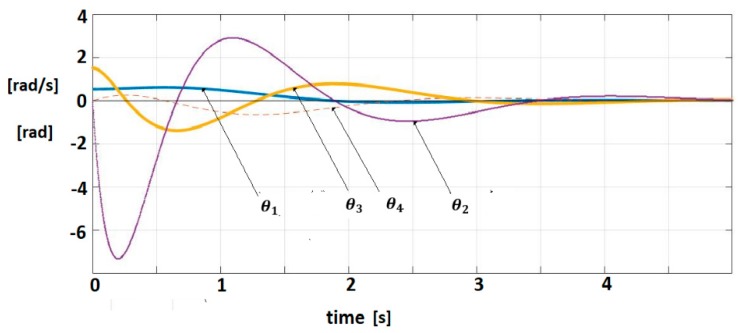
Fractional-order variable trajectories for FOM system with IOM sensor.

The pairs are (A,b), (A,c) controllable, respectively observable. The IOM sensor output without delays is given by (7). A control law (Equation (13)) (for $y_{ref}\left(t\right))$ = 0) with k=200 is applied. This control verifies the sector constraint (Equation (11)) with $\sigma =5\times 10^{-3}$. The matrix R was considered as, R=diag(3,3,3,3) where $A_{1}=A-R$ is Hurwitz stable. The vector $q=0.05\times {\left[1111\right]}^{T}$ and a matrix P were inferred with $\lambda_{\max\left(P\right)}=0.725$. The polar plot of $c^{T}{\left(j\omega I-A_{1}\right)}^{-1}b$ is shown in Figure 6. The closed-loop system satisfies the frequential criterion (Equation (17)), condition (18) is verified for ||Q||=14.5, ρ=2.17, MATLAB/SIMULINK and techniques based on the Mittag-Leffler functions are used for the simulation [1,2]. Figure 7 shows the trajectories of fractional order variables.

The steady state behavior can be analyzed by using by transfer function of Equations (65)–(67) (linearized model)

(71) $$H_{EX}\left(s\right)=\frac{\theta \left(s\right)}{u\left(s\right)}=\frac{900}{s^{2}+44s^{\frac{1}{2}}+1560s+530}$$

The control law (Equation (13)) can be rewritten as a PD control

(72) $$u_{PD}=-k_{1}z_{1}-k_{3}D^{\frac{1}{2}}\left(D^{\frac{1}{2}}z_{1}\right)=-k_{1}z_{1}-k_{3}{\dot{z}}_{1}$$

and the controller transfer function will be

(73) $$H_{C\left(PD\right)}\left(s\right)=k_{1}+k_{3}\;s\;=\;200\left(1+s\right)$$

For the PD^0.5^ control law, we have

(74) $$H_{C\left(PD^{0.5}\right)}=k_{1}+k_{2}s^{0.5}=200\left(1+s^{0.5}\right)$$

The steady error can be inferred from Equations (71)–(73) as [21,32,33]

$$e_{s\left(PD\right)}=e_{s\left(PD^{0.5}\right)}=0.0042$$

**Figure 8:**
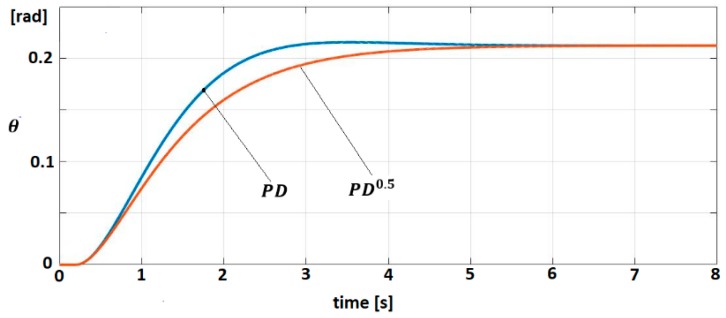
Trajectory θ(t) for PD^0.5^ and PD

The behavior of the linearized model (Equation (68)) for both control laws (Equations (73) and (74)) is studied. The trajectories of angular position θ for target signal $\theta_{targ}=\frac{\pi }{6}$ are shown in Figure 8.

#### 3.1.2. EXHAND with Delay

The sensor dynamics are

(75) $$K_{s1}\;z_{1}\left(t\right)=y_{1}\left(t\right)$$

(76) $$K_{s2}\;z_{2}\left(t-\tau \right)=y_{2}\left(t\right)$$

where τ∈[−0.1;0].  Substituting Equations (74) and (75) into (68) and using the control law (Equation (70)), yields

(77) $$J\;\ddot{\theta }\left(t\right)=-c_{\nu }D^{\beta }\theta \left(t\right)-\left(c_{e}+K_{s1}k_{1}\right)\theta \left(t\right)-c_{d}\;\dot{\theta }\left(t\right)-K_{s2}k_{2}\;\dot{\theta }\left(t-\tau \right)+k_{2}bc_{2}^{T}D^{\beta }\theta \left(t-\tau \right)+mg\sin\theta$$

with initial conditions

(78) $$\theta \left(t\right)=\frac{\pi }{3},\;\dot{\theta }\left(t\right)=-1,\;t\in \left[-0.1;0\right]$$

**Figure 9:**
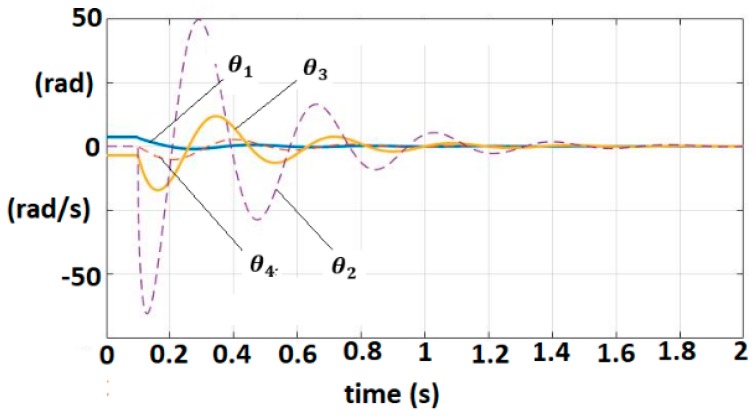
Fractional-order variable trajectories for FOM EXHAND with delay.

The delay component of the dynamic model is defined by τ=0.1 s. The controller parameters are selected as $k_{1}=200,\;k_{2}=15$ that satisfy Equations (29)–(31) by employing the same parameters for q, R as in the previous example. The evolution of the fractional order variables is shown in Figure 9.

#### 3.1.3. EXHAND with Delay and Observer

**Figure 10:**
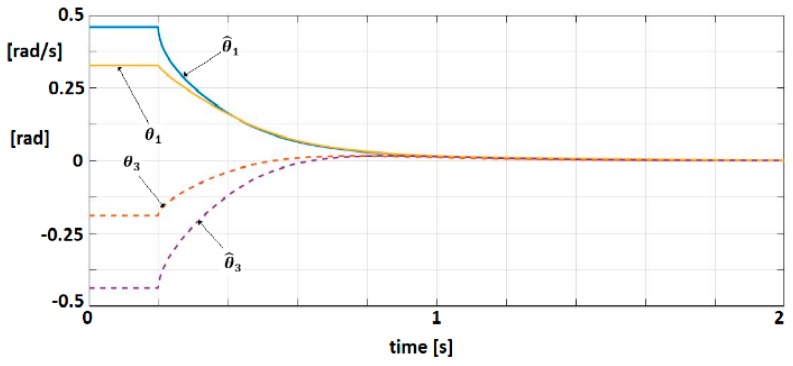
Trajectories of position and velocity for the system and observer.

An observer (Equations (49)–(51)) with $L_{1}=L_{2}=\;\left[1.5\;1.5\;1.5\;0\right]{\;}^{T}$ is associated to the linearized dynamic model. The matrix R=diag(1 1 1 1) verifies the condition as $\left(A_{L}-R\right)$ to be Hurwitz matrix. For $q=\left[0.5\;0.5\;0.5\;0.5\right]{\;}^{T}$, solution of $P_{1}$ is obtained with $\lambda_{\max\left(P_{1}\right)}=0.0085$. A control law (55) with $k_{1}=20,\;k_{2}=8.5,\sigma =0.05$ were selected. Equations (57)–(61) are easily verified. Figure 10 shows the trajectories of physical significance variables, position and velocity, for the system and observer.

### 3.2. IHRG Experimental Platform

The IHRG is an exoskeleton that supports the human hand and hand activities by using a control architecture for dexterous grasping and manipulation. IHRG is a medical device that acts in parallel to a hand in order to compensate for lost function. It is easy to use and can be a helpful tool in the home [16,34].

**Figure 11:**
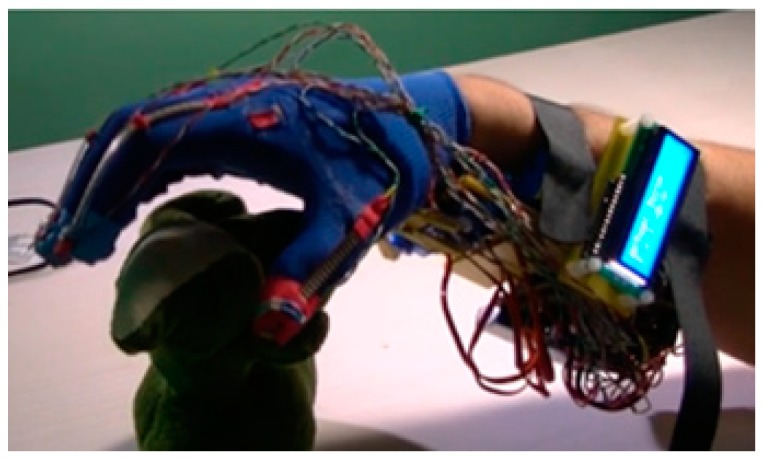
IHRG-human hand exoskeleton.

**Figure 12:**
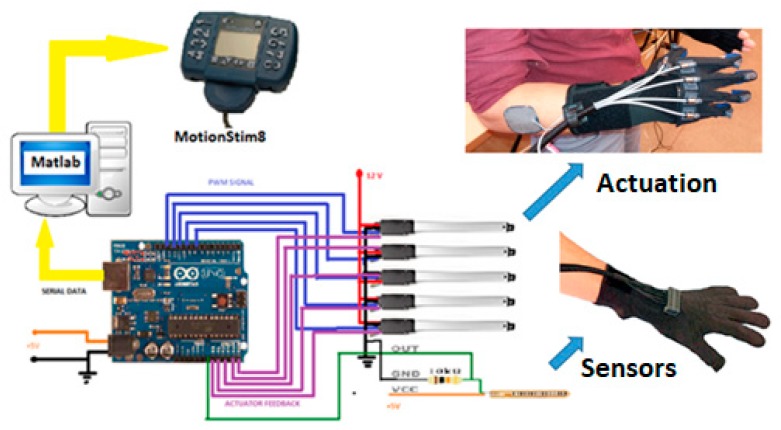
IHRG general architecture.

The mechanical architecture consists of articulated serial elements of which design covers functional and anatomic finger phalanges. The glove is created by a thin textile that represents an infrastructure suitable for actuation wires and sensors. A distributed actuation system is used for implementing the operations of the hand. An Arduino Flex Sensor network (with zeroth order sensors) is used to control the motions. An Arduino Mega 2560 hardware platform determines the movement of the glove’s actuators for exercises like opening or closing of the fingers (Figure 11 and Figure 12).

**Figure 13:**
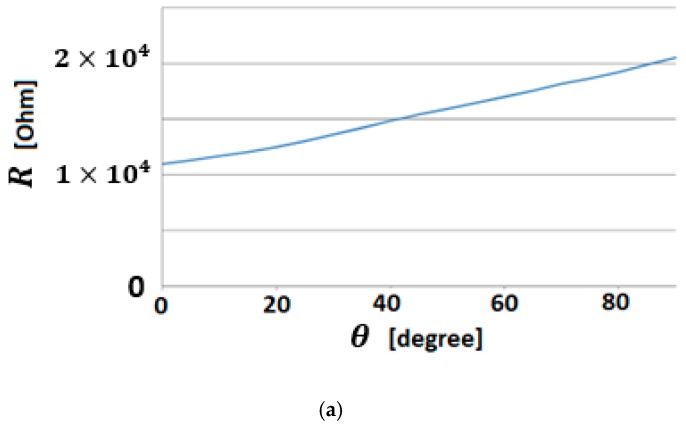

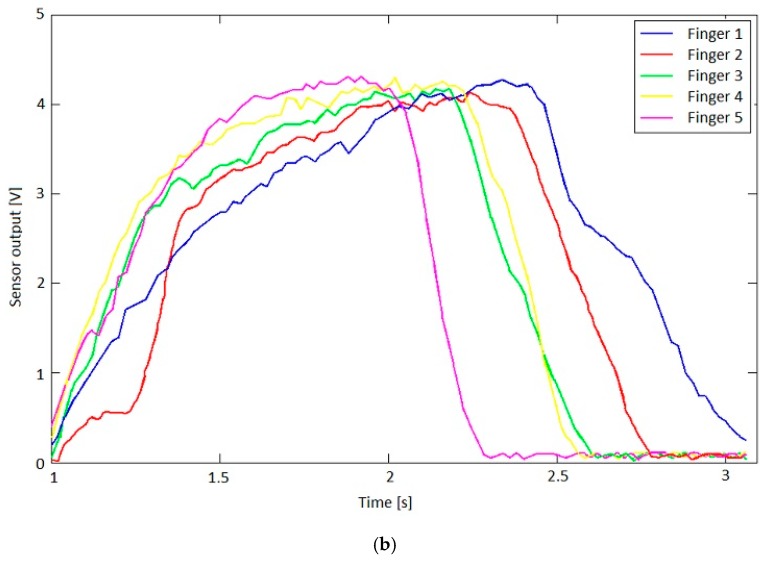
(**a**) Bending sensor characteristics; (**b**) Output values of the bending sensors.

All the movements of the hand are controlled by the software of the hybrid IHRG system, which was developed in MATLAB and Simulink. The performance of each patient following the exercises program can be recorded by the same software. The control system of Control System 1 is implemented. In Figure 13 are shown the sensor signals during an open-close-open hand exercise.

## 4. Discussion

I. We designed, built, and tested an intelligent haptic robotic glove for the rehabilitation of the patients that have a diagnosis of a cerebrovascular accident. The glove is created by a thin textile in order to have a comfortable environment for the grasping exercises. This thin textile creates an infrastructure suitable for wire actuation and sensors. This exoskeleton architecture ensures the mechanical compliance of human fingers. The driving and skin sensor system is designed to determine comfortable and stable grasping function. The dynamics of the exoskeleton hand are modeled by fractional order operators. To our knowledge, this paper is the first paper in which the interaction between biological systems (human hand) and mechanical associated components (exoskeleton) is analyzed by fractional order models. These new models are used to develop a class of algorithms for the control of the stable grasping function. The control systems are based on the physical significance variable control that are generated by sensor classes implemented in the system. These sensors are also modeled as operators with delays. The paper proposes control solutions and determines the criterions for controller parameter tuning for several classes of models. The observer techniques are also discussed and implemented. The quality and the stability of motion, are analyzed by Lyapunov methods and techniques that derive from Yakubovici-Kalman-Popov Lemma.

Despite of the model complexity, the control systems are very simple, and the controllers are easily implemented in an Arduino Mega 2560 hardware platform. There were many advantages for using this platform since this hardware board has ports for PWM signals that are useful to be sent to the actuators and ports for reading the signals coming from the bending sensors.

In order to help patients to follow an after-stoke recovery program, the system uses a set of predefined rehabilitation exercises like open the hand, close it, try to grab an object or simple wave. The system is very easy to use at home, with minimal training. The predefined rehabilitation set of exercises was created to be used.

**Figure 14:**
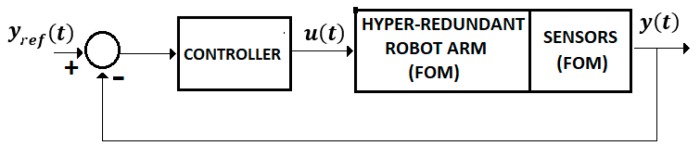
Control system with FOM sensors.

II. The control systems discussed in the previous sections are focused on the control problems of the IHRG system, where the EXHAND model is described by FOM operators and the sensor system is based on zeroth order sensors. These control solutions can be also used for a larger class of complex systems as hyper-redundant systems, that use complex FOM sensors (Figure 14).

**Figure 15:**
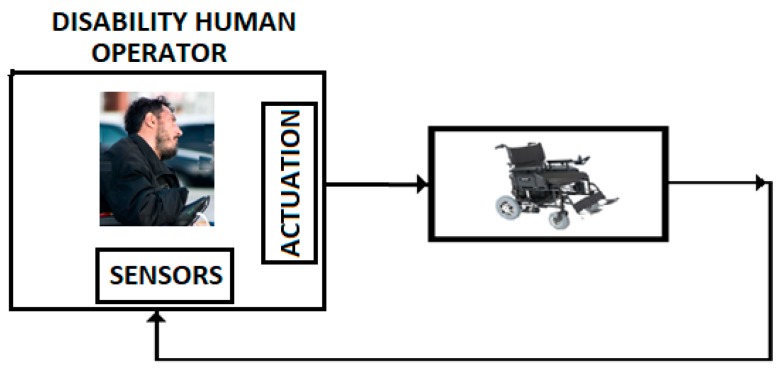
Control with disability human operator.

III. In addition, we consider that control systems discussed in the previous sections can be applied to a class of control problems associated to the persons with disabilities. Figure 15 presents a wheelchair control system for this class of persons. In this case, the human operator is represented by the persons with hemiparesis/hemiplegia, with motor restriction (arm or leg-emphasized hemiparesis) and with serious brain damage [35],

$$H_{h}\left(s\right)=k_{h}\frac{e^{-\tau s}}{s^{\beta }}$$

The transfer function of this human operator has a model that corresponds to a time delay fractional order model with time constant τ and fractional order β. These parameters are determined by the characteristics of the damaged brain, viscoelastic properties of the atrophied muscles, and propagation time along the nervous terminals.

We consider that these models can be studied by using the techniques developed in this paper.
